# Identifying baseline immune-related biomarkers to predict clinical outcome of immunotherapy

**DOI:** 10.1186/s40425-017-0243-4

**Published:** 2017-05-16

**Authors:** Sacha Gnjatic, Vincenzo Bronte, Laura Rosa Brunet, Marcus O. Butler, Mary L. Disis, Jérôme Galon, Leif G. Hakansson, Brent A. Hanks, Vaios Karanikas, Samir N. Khleif, John M. Kirkwood, Lance D. Miller, Dolores J. Schendel, Isabelle Tanneau, Jon M. Wigginton, Lisa H. Butterfield

**Affiliations:** 1 Department of Hematology/Oncology, Tisch Cancer Institute, Icahn School of Medicine at Mount Sinai, S5-105, 1470 Madison Avenue, Box 1128, New York, NY 10029 USA; 2 Head of Immunology Section, University of Verona, Piazzale Le L. A. Scuro, 10, Verona, Italy; 3 Immodulon Therapeutics Ltd, Stockley Park, 6-9 The Square, Uxbridge, UK; 4 Princess Margaret Hospital/Ontario Cancer Institute, RM 9-622, 610 University Ave, Toronto, ON Canada; 5 University of Washington, Tumor Vaccine Group, 850 Mercer Street, Box 358050, Seattle, WA 98109 USA; 6 INSERM - Cordeliers Research Center, Integrative Cancer Immunology Laboratory, 15 rue de l’Ecole de Médecine, Paris, France; 7 CanImGuide Therapeutics AB, Domkyrkovägen 23, Hoellviken, Sweden; 8 Duke University Medical Center, 308 Research Drive, LSRC, Room C203, Box 3819, Durham, NC 27708 USA; 9 Roche Innovation Center Zurich, Wagistrasse 18, Schlieren, Switzerland; 10 Georgia Cancer Center, Augusta University, 1120 15th Street, CN-2101A, Augusta, GA 30912 USA; 11 University of Pittsburgh, Hillman Cancer Center-Research Pavilion, 5117 Centre Avenue, Suite 1.32, Pittsburg, PA 15213 USA; 12 Wake Forest School of Medicine, 1 Medical Center Blvd, Winston Salem, NC 27157 USA; 13 Medigene Immunotherapies GmbH, Lochhamer Strasse 11, Planegg-Martinsried, Germany; 14 ImmunID, 7 Parvis Louis Néel, BP50 Grenoble, France; 15 MacroGenics, Inc., 9704 Medical Center Drive, Rockville, MD 20850 USA; 16 Department of Medicine, Surgery and Immunology, University of Pittsburgh Cancer Institute, 5117 Centre Avenue, Pittsburgh, PA 15213 USA

**Keywords:** Cancer immunotherapy, Tumor microenvironment, Biomarkers, Baseline immunity, Immune checkpoint inhibitors

## Abstract

As cancer strikes, individuals vary not only in terms of factors that contribute to its occurrence and development, but as importantly, in their capacity to respond to treatment. While exciting new therapeutic options that mobilize the immune system against cancer have led to breakthroughs for a variety of malignancies, success is limited to a subset of patients. Pre-existing immunological features of both the host and the tumor may contribute to how patients will eventually fare with immunotherapy. A broad understanding of baseline immunity, both in the periphery and in the tumor microenvironment, is needed in order to fully realize the potential of cancer immunotherapy. Such interrogation of the tumor, blood, and host immune parameters prior to treatment is expected to identify biomarkers predictive of clinical outcome as well as to elucidate why some patients fail to respond to immunotherapy. To approach these opportunities for progress, the Society for Immunotherapy of Cancer (SITC) reconvened the Immune Biomarkers Task Force. Comprised of an international multidisciplinary panel of experts, Working Group 4 sought to make recommendations that focus on the complexity of the tumor microenvironment, with its diversity of immune genes, proteins, cells, and pathways naturally present at baseline and in circulation, and novel tools to aid in such broad analyses.

## Background

Immunogenic cancers persist despite the presence of tumor-specific adaptive immune responses through intricate interactions between tumor cells and the host immune response within the tumor microenvironment (TME). The presence of pre-existing local adaptive immunity has been associated with positive outcomes in a variety of malignancies [1, 2], and as such, initiatives to overcome immune escape and subsequently enhance immune function have been at the forefront of the cancer immunotherapy field. Several recent efforts have invested in understanding how the immune cell context within the TME can act either as a predictive or prognostic factor in response to a given therapy, or guide combination partner selection and improve clinical outcomes [3]. As data from ongoing clinical trials with checkpoint inhibitors and other immuno-oncology drugs become more widely available [4, 5], understanding the complex relationships between immune and tumor cells within the tumor tissue promises to help us understand how to better convert non-inflamed to inflamed tumors and address immune escape [6, 7].

With regard to prognostic biomarkers, compelling evidence from multiple studies has revealed that infiltration by leukocyte subsets such as CD8+ and CD45RO+ memory T cells with specific cytokine signatures (e.g., dense infiltration by immunohistochemistry or a cytotoxic gene profiling) and perhaps B cells too, is linked with favorable outcome in a variety of cancers, regardless of potential immunotherapeutic intervention [1, 8–11]. In a landmark colorectal cancer study, adaptive immune cell infiltration was shown to have a prognostic value superior to the classical extension and invasion tumor criteria [1, 12]. A resulting “immunoscore” quantifying the density of CD3+ and CD8+ T cells in the tumor center and its invasive margin was proposed as a novel immune classification colorectal tumors [1, 13]. Similar information has been available in other cancers such as melanoma where tumor infiltrating lymphocytes have been recognized to be of prognostic and predictive utility for 20 years [14], which have been reinforced by the findings of The Cancer Genome Atlas [15]. Involvement of other subsets, such as regulatory T cells (Treg) and macrophages, has been investigated, showing that they can confer either good or poor prognosis depending on context [9, 16]. Immune signatures associated with immune-mediated tissue destruction (e.g., genes, proteins, or cells related to cytotoxicity), or conversely genetic or histological signatures associated with immune suppression, will influence the immune contexture. Thus, a continuum exists, tilting the balance towards either tumor cell growth or elimination, governed by pre-existing immunosurveillance [17]. This context is where treatment with immunomodulatory drugs act, helping to further shift the scale against cancer.

The remarkable clinical successes of multiple new immunotherapeutic strategies in the last 6 years have largely relied on targeting suppressive mechanisms affecting T cells. This is particularly the case for checkpoint inhibitors, such as the US Food and Drug Administration (FDA)-approved antibodies targeting cytotoxic T lymphocyte-associated protein 4 (CTLA-4) [5] and programmed cell death protein 1 (PD-1) and its ligand, PD-L1 [4, 18, 19]. The identification of predictive biomarkers is likely to be the most fruitful if we can understand pre-existing antitumor immune profiles, by interrogating the TME for T cells [20], the antigens they target including neoantigens, and suppressive intrinsic and extrinsic factors affecting them. The best studied predictive biomarker of immunotherapy is the PD-1/PD-L1 inhibitory axis, where tumor expression of PD-L1 by some tumors may correlate with better clinical response to treatment with anti-PD-1 or anti-PD-L1 antibodies [21]. Co-localization of PD-L1 expression within an inflamed TME suggests that PD-L1 expression is upregulated in the setting of an endogenous anti-tumor immune response [22, 23]. PD-1/PD-L1 blockade appears to result in enhancement of the localized inflammatory response with further PD-L1 upregulation in responding patients [20, 23]. Moreover, the phenotype of tumor-antigen specific infiltrating lymphocytes within the TME suggests that the majority of these cells reside within the PD-1 positive fraction [24–27]. The demonstrated clinical impact of checkpoint inhibition on patient outcomes notwithstanding, absolute predictors of a tumor response based on immune infiltration have yet to be defined. Accumulating exceptions such as lack of response to treatment in some patients, the incomplete correlation between PD-L1 expression and clinical effectiveness of PD-1 blockade [4, 28, 29], and the counterexamples in renal cell carcinoma in which the presence of T cells is generally associated with poor outcome [30] indicate that a more comprehensive profiling of local immune cells and of their function is warranted.

Efforts to profile tumor-infiltrating immune cells however often have inherent limitations in sample availability and technological capability, thus restricting investigations into the local immune response. New tools equipped to ask more complex questions have led investigators to revisit old observations as well as to pursue new lines of inquiry from peripheral blood as well. For T cells, considered as the major mediators of antitumor activity, efforts to characterize their specificity are critical, from defining shared antigens to identifying mutation-derived neoepitopes. Accordingly, the use of various tests of T cell specificity, functionality, clonality, or diversity may inform us on spontaneous tumor immunogenicity and provide a surrogate for potential antitumor effector function. For B cells, early autologous typing studies and advanced microarray profiling of cancer patient sera have demonstrated that circulating antibodies to tumor-derived antigens arise in response to cancer development or progression [31, 32]. While there is still no routinely used test for circulating antibodies with predictive value in cancer, some have proposed using serum antibodies to tumor antigens such as p53 or MUC1 as diagnostic markers [33], and others such as NY-ESO-1 as prognostic indicators of higher grade and larger tumor mass [34].

Other peripheral markers that may reflect informative aspects of the TME remain to be explored. For example, preclinical evidence supports a key role for myeloid-derived suppressor cells (MDSC) in the control of cancer progression, either by inhibiting adaptive and innate immunity against cancer or by affecting basic steps of tumor development, such as neoangiogenesis, local and metastatic spread, and cancer stemness [35, 36]. MDSC presence and frequency in the blood of tumor patients might represent a novel and simple biomarker to monitor clinical outcome and response to therapy [37]. However, the specificity for cancer is not absolute since MDSC can also expand under noncancerous conditions, such as sepsis, bacterial, viral, and parasitic infections, autoimmunity, and in aging individuals [35, 36].

In addition to local and peripheral tumor immunity, host-related factors, including single nucleotide polymorphisms (SNP), also contribute to the still elusive “immunocompetence” status of cancer patients toward their tumor. The integration of powerful technologies such as genome-wide association studies (GWAS), multiplex immunohistochemistry (IHC), and mass cytometry is expected to aid in our interpretation of such increasingly complex questions at the genetic, molecular, and cellular levels from which we might better predict therapeutic benefit. Collectively, pre-existing tissue and peripheral immune-related biomarkers in the context of host factors are poised to inform researchers and clinicians about the immune competence and likelihood of response in cancer patients undergoing immunotherapy. Here, we will discuss these aforementioned aspects of clinical outcome prediction based on measures of baseline immunity in the TME and in blood, and make recommendations for the future of this field.

## Biomarkers at the tissue site prior to treatment

### How the tumor microenvironment at a cellular level determines therapeutic approaches

Prognostic markers evaluating individual patient outcome, such as recurrence of disease or death, independent of therapy, range from simple measures, including stage of disease based on tumor invasion, to progressively more comprehensive indicators encompassing the biological complexity of the disease [12, 38]. Indeed, the evolution of cancer is greatly influenced by the complex milieu in which it develops, accommodating intricate tumor-cell interactions within the host microenvironment including a vast catalogue of cells, vessels, cytokines, and chemokines. Histological analysis of human tumors has highlighted the importance of tumor immune infiltrates including macrophages, DC, polymorphonuclear cells, natural killer (NK) cells, B cells, and T cells, revealing a broad patient-to-patient diversity [13]. Among an increasing variety of investigations supporting the relevance of the differential presence of immune system components in determining the evolution of cancer [39], a predominant theme based on direct human observations collectively suggests that high densities of TIL correlate with improved clinical outcome [13]. The correlation between a robust lymphocyte infiltration and better patient survival has been well documented in melanoma, ovarian, head and neck, breast, urothelial, colorectal, lung, hepatocellular, esophageal cancer, and brain metastases [9, 40]. The majority of studies observed that high densities of CD3+ T cells, CD8+ cytotoxic T lymphocytes (CTL), and CD45RO+ memory T cells are associated with a longer disease-free survival (DFS) and/or improved OS. Thus, the role of the adaptive immune response in controlling tumor progression is becoming increasingly appreciated. Although tumor-infiltrating lymphocytes are often dysfunctional, their presence indicates that there is no systemic inhibition of recruitment. These prognostic immune parameters have been comprehensively described as the immune contexture, and define a novel paradigm for cancer. Chemo-attraction and adhesion were shown to play critical roles in determining the density of intra-tumoral immune cells. Expression of specific chemokine signatures correlated with differing densities and spatial localization of T cell subpopulations within tumor regions, and with specific TCR repertoires predicting patient survival [41]. Local proliferation of CD8+ T cells mediated through the expression of *IL15* was also demonstrated as a mechanism leading to increased CTL density [42]. High expression levels of these immune-related genes were associated with prolonged disease-free survival (DFS) in patients with colorectal cancer, and long-term OS correlated with these immune gene signatures [41]. Similar gene expression profiles were also observed in additional studies [43–48].

An international consortium was organized to validate and promote the use of Immunoscore in routine clinical settings [49, 50]. Immunoscore has a prognostic value in early-stage patients [51], as well as in late-stage patients such as patients with brain metastases [40]. To be used globally in a routine manner, evaluation of a novel marker should be: routine, feasible, simple, rapid, robust, reproducible, objective, specific, quantitative, standardized, powerful, and preferentially pathology IHC-based. Immunoscore has the potential to fulfill these key criteria. In addition, Immunoscore provides a tool for novel therapeutic approaches, including immunotherapy [4, 5, 18, 19]. The findings of this international consortium may result in the implementation of the Immunoscore as a new component for the classification of cancer, designated TNM-I (TNM-Immune).

### Multiplex IHC in clinically annotated material

Initial reports defining the clinical impact of tumor infiltration by immune cells, such as the Immunoscore, have recognized that while the high density of memory CD8+ T cells may predict long-term survival of colon cancer patients, it is equally important to address the location and functional differentiation of such cells, whether inside the tumor itself or in surrounding stromal areas [1, 9, 52]. Beyond localization, evidence is mounting that solid tumors harbor a variety of immunocytes beyond T cells that may be associated with good or poor outcome. Therefore, defining only one or two immune markers is unlikely to be sufficient, and multiparametric approaches are needed to comprehensively assess immune profiling of cells within the tissue architecture from baseline.

Recent advances in tumor tissue multiplex IHC technologies aim to provide insights into the nature of tumor immune infiltration with respect to the type, number, and qualitative characteristics of the immune cells present, as well as their interactions with the tumor and stromal cells as a correlate to disease progression and prognosis. Multiplex IHC offers the unique opportunity to dissect the dynamic interactions between immune cells and the TME. However, undertaking such multiparametric analyses has been met with various technological and biological challenges [53]. For instance, multiplexing applications have been limited by which antibodies can be combined without cross-reactivity, insufficient specificity of some reagents, and confounded by spatial co-expression of some antigens that may interfere with precise interpretations of results. These problems are compounded by the limited availability of overlapping chromogenic agents. Despite these hurdles, the use of fluorescently-labeled antibodies offers improved multiplexing capabilities, and advances are being made to reuse fluorescent or chromogen-stained slides multiple times for consecutive analyses on the same tissue [54, 55].

IHC assessments have generally utilized two to three markers simultaneously, with additional staining undertaken on separate serial sections if more markers were required [56, 57]. Most of the duplex or triplex IHC assays to date employ chromogenic tools since this is a well-established approach in visualizing several antigens. Tumeh et al. reported an increased CD8+ T cell density in post-treatment serial biopsies from responding melanoma patients treated with pembrolizumab [20]. Furthermore, additional functional characterization is usually acquired by molecular profiling in serial sections. For instance, biopsies of patients responding favorably to checkpoint inhibition show an increased number of proliferating CD8+ T cells associated with increased levels of PD-L1 expression as assayed by IHC and an increased IFNγ signature as determined by gene expression profiling [23, 58]. Moreover, a high tumor-infiltrating lymphocyte (TIL) presence and PD-L1 expression determined by IHC correlated with IFNγ-producing immune cells identified by qRT-PCR of laser micro-dissected specimens [59].

To characterize the T cells in the TME for their specificity, technologies employed thus far with some degree of success utilize either the recognition of antigen-specific T cells by in situ major histocompatibility complex (MHC) class I tetramer staining or TCR Vβ repertoire analysis [60, 61]. The wider applicability of the former has been rather limited due to specificity constraints mostly against melanoma antigens. With respect to the latter, this approach has enabled a positive association of PD-1 expressing T cells and PD-L1 expressing cells in tumors determined by IHC with a more restricted Vβ-chain usage as a response to pembrolizumab, highlighting the potential utility of this approach [20]. Multiparametric IHC approaches are now being utilized together with efforts to characterize the mutational spectrum of the underlying TME in order to characterize the immune responses they elicit, as discussed next [60, 61].

Investing in advancing multiplex IHC technologies utilizing fluorescence-, chromogen-, or heavy metal-labeled antibodies that can maximize the use of the limited material available in a clinical setting could ensure a “true” overlay of different immune markers and determination of marker co-expression. Coupling this IHC technology with mutational profiling and gene expression patterns could offer a more comprehensive understanding of the TME and promises a future whereby immune biomarkers could inform therapeutic choices in order to improve clinical outcome of cancer immunotherapy treatments.

### Gene expression at the tumor site

Since the introduction of expression microarray technologies, genes with specialized roles in immune cell biology have been repeatedly observed to be highly expressed components of tumor expression profiles of some patients. Based on the coordinate expression among these genes [62–66], their positive correlation with histological measurements of TIL [15, 58, 62, 63], and their enriched expression in immune cell lineages [62], it is now widely accepted that these genes reflect the relative abundance of various populations of tumor infiltrating leukocytes. Consistent with this hypothesis, numerous robust and reproducible associations between immune gene signatures in solid tumors and clinical outcomes have been reported. In aggressive subtypes of breast cancer, gene signatures believed to reflect anti-tumor involvement of T cells (CTL and Th cells) or B cells (namely plasma cells) have been shown to exhibit highly significant positive associations with OS and recurrence-free survival of patients [62–64, 67–70], as well as pathologic complete response in the neoadjuvant setting [71–75]. In colorectal cancers, the expression of genes believed to underlie CTL, Th cells, and B cells has been associated significantly with prolonged recurrence-free survival [76]. The tumor microenvironment and Immunoscore were shown to be critical determinants of dissemination to distant metastases [77]. Similarly, prolonged OS and distant metastasis-free survival has been associated with the high expression of genes believed to reflect T cell, B cell, and natural killer (NK) cell involvement in metastatic melanoma [15, 78]. In most instances the prognostic attributes of these immune gene signatures remain significant in multivariate models, indicating that they provide prognostic information not captured by conventional prognostic factors such as tumor stage, grade, size, and nodal status [62, 69, 71, 73, 78].

In the context of immunotherapy, the predictive potential of immune genes has been elucidated recently. In a phase II clinical trial comparing ipilimumab doses in metastatic melanoma, Ji et al., discovered that T cell-related genes were significantly over-expressed in pre-treatment biopsies of subjects with ipilimumab clinical activity [58]. Among the genes were T cell surface markers (CD8A, CD3, CD2, CD277, CD27, and CD38), cytotoxic factors (PRF1 and GZMB) and tissue rejection-related cytokines and chemokines (CXCL9, CXCL10, CXCL11, CCL4, and CCL5), all of which have been observed as central components of previously described prognostic and therapy-predictive immune gene signatures [62, 63, 71, 72, 75]. In a phase II trial of recombinant MAGE-A3 protein in combination with different immune stimulants in metastatic melanoma, Ulloa-Montoya et al. discovered an immune-related gene signature that was associated with clinical benefit in melanoma patients [79]. Similar to that discovered by Ji et al., key genes of this signature included CD8A, CD3D, CCL5, CXCL9, CXCL10, CXCL2, GZMK, and other genes related to T cell function and immune signaling. Intriguingly, the same gene signature significantly predicted favorable DFS in non-small cell lung cancer (NSCLC) patients treated with MAGE-A3 (plus AS02 immune stimulant) but not those treated with placebo [79].

Together, these observations support the notion that gene expression-based correlates of immune involvement could hold valuable clinical utility for a number of prognostic and therapy-predictive applications. However, to date, mRNA-based diagnostics that quantify immune involvement in tumors do not exist. Multi-gene diagnostics that simultaneously measure mRNA transcripts of multiple genes represent a class of In Vitro Diagnostic Multivariate Index Assay (IVDMIA) that has in recent years gained wide clinical acceptance for the diagnosis and stratification of patients into risk groups to guide therapeutic decisions [80, 81]. Such diagnostics are currently being developed on platforms engineered for high sensitivity and specificity of mRNA detection and multiplex capability such as real-time quantitative PCR (Oncotype DX test), expression microarrays (MammaPrint test), and the NanoString n-Counter platform (Prosigna test). Unlike other clinical biomarkers that rely on cell type-specific detection, multi-gene tests typically quantify gene expression from whole tumor specimens. Thus, a multi-gene IVDMIA might represent a suitable context for the diagnostic development of immune gene signatures. However, immune assessment from whole tumor fragments carries potential advantages and disadvantages compared to conventional IHC-based approaches. Immune analysis of whole tumor fragments might provide a more representative sampling of the distribution of immune cells throughout a tumor as compared to a conventional two-dimensional tumor section. Also, the quantification of a panel of immune genes may have the advantage of objectivity and cost-effectiveness as compared to more subjective strategies for quantifying proteins in multiplicity by conventional IHC. By contrast, transcript analysis in tumor fragments could be confounded by admixed cell types, where the diagnostic signal may be obscured by transcripts that are not necessarily specific to the target cell population (i.e., transcripts expressed by both cancer and noncancerous cells). New computational methods, however, such as ESTIMATE [82] and CIBERSORT [83, 84] that utilize cell-specific gene expression signatures to infer relative fractions of immune and stromal cell populations from whole tumor gene expression profiles are making progress toward this limitation. These methods employ deconvolution and require next-generation sequencing (NGS) of the tumor sample.

### How the tumor microenvironment at a genetic level determines therapeutic approaches

The ability to predict response to treatment is important in all cancer therapies but particularly germane to newly-approved agents where toxicity can be severe, and cost plays a major role in treatment decisions. Small molecule inhibitors of constitutively active tyrosine kinases have radically changed the treatment paradigm for lung cancer and chronic myelogenous leukemia. The importance of genetic mutations in the efficacy of immunotherapy has only recently been highlighted and these functional mutations are likely to become an integral part of tumor characterization at baseline for immunogenicity.

Genetic mutations in tumors are associated with an enhanced response rate to therapy with agents that target CTLA-4 and PD-1 [85, 86]. The highest response rates to nivolumab and pembrolizumab are seen in Hodgkin lymphoma and microsatellite unstable (MSI high) colon cancer [87–89]. In Hodgkin lymphoma, PD-L1 overexpression is the result of enhanced transcription driven both by JAK2, PD-L1, and PD-L2 overexpression caused by gene amplification on the chromosome 9 locus that encodes these genes. In MSI high colon cancer, mutations in the DNA repair mechanisms predispose to colon cancer but also produce high rates of mutations in other genes that can function as tumor antigens. The efficacy of immune checkpoint blockade is also high in patients with genetic mutations leading to the generation of peptides that drive the expansion of T cells that are either pre-existing or that can be generated in response to bacterial, viral, or other immune stimuli [85]. The existence of tumor reactive T cells in turn results in the production of cytokines such as TNFα and IFNγ that upregulate the expression of PD-L1 in the TME. As expected, therefore, PD-L1 positive tumors have response rates significantly higher than PD-L1 low or negative tumors. The studies of pembrolizumab in lung cancer separated therapeutic effects into three groups based on the level of PD-L1 staining: those with 50% or greater positivity in tumor, 1-49% positive, and less than 1% PD-L1 positive. The response rates were 45.2, 16.5, and 10.7%, respectively. Even higher response rates are observed in patients with no prior therapy in all three groups [86]. This observation suggests that prior therapy may blunt the ability of the immune system to produce tumor regression and highlights the need to introduce immunotherapy earlier in the course of disease to maximize benefit in inoperable disease; this also forms the foundation of the rationale for adjuvant applications of these agents in operable disease at high risk of postoperative relapse.

PD-L1 expression identifies tumors that have an increased likelihood of response to immune checkpoint blockade, however 10–20% of the PD-L1 negative or low tumors also respond [90]. This suggests that in some tumors the T cells exist to make the tumors regress but that their numbers are insufficient to drive PD-L1 expression in the tumor. It may be possible to determine the patients whose tumors will become positive for PD-L1 expression through the use of IFNγ administration. In this regard it is interesting to remember the results of the adjuvant use of IFNγ in patients with melanoma. In the randomized trial of adjuvant IFNγ, there was both increased recurrence rate, and earlier recurrence among patients allocated to IFNγ therapy compared with placebo [91]. It is possible that the IFNγ production caused PD-L1 upregulation in the tumor and subsequent enhanced tumor growth as a result of the resistance induced by PD-L1 expression.

The outstanding results of nivolumab in Hodgkin lymphoma may be due to constitutive PD-L1 expression. Characterization of other tumors with similar amplifications on chromosome 9 may identify tumors of other histological profiles with an enhanced rate of tumor response.

### Tumor antigens, mutational load and neoantigens

Identifying whether the presence of activated effector T cells in the TME relates to T cells with a given antigen specificity is a priority, given mounting evidence that the tumor mutational load contributes to tumor immunogenicity and eventual destruction [85, 86]. Understanding the specificity of T cells in tumors at baseline may therefore be a key to immunotherapy’s success. However, identifying immune responses to antigens unique to tumors and not expressed on normal tissue can be cumbersome, even when targeting known shared tumor antigens such as NY-ESO-1. An alternative approach is to use mutational burden in tumors as a proxy for the presence of T cell epitopes derived from neoantigens, which are mutated peptides that arise in tumors but are not present in the normal genome [92]. The identification of novel neoantigens has recently become more feasible with the use of whole exome sequencing. Next generation sequencing of tumors to identify mutations and the use of computer algorithms to identify mutated peptides that bind to MHC molecules can help to select the appropriate targets for T cell enhancement. Frameshift mutations in microsatellite-unstable tumors showed genetic evidence of immunoediting, contained higher densities of Th1 cells, effector-memory T cells, in situ proliferating T cells, and inhibitory PD-1/PD-L1-expressing cells, had a high Immunoscore, and were infiltrated with mutation-specific cytotoxic T cells [93]. Recent findings support accumulating data that it is not singular mutations that predict patients’ clinical outcome, but rather the presence of a high number of mutations and a global T cell response in the TME [94].

Multiple types of cancer antigens have been characterized, including neoantigens such as those encoded by mutations and viral antigens, self-proteins that are either overexpressed or usually not expressed in most of the adult body (e.g., cancer testis antigens), and tissue-specific gene products in which the cancer affects a tissue or cell type not essential for the life of the patient (e.g., B cells, melanocytes, or the prostate). Furthermore, antigenic peptides do not simply correspond to fragments of conventional proteins, but rather result from aberrant transcription, incomplete splicing, translation of alternative or cryptic open reading frames, or post-translational modifications. Proteasome peptide splicing also represents another mechanism that increases the diversity of antigenic peptides presented to T cells [95]. Antigenic peptide processing is a complicated process that involves a multitude of human leukocyte antigens [92]. Cancer-associated aberrant protein O-glycosylation can modify antigen processing and immune response [96] and MHC class I–associated phosphopeptides are the targets of memory-like immunity. Results point to a role for phosphopeptide-specific immunity as a component of tumor recognition and control [97]. Thus, beyond exome sequencing and point mutations, various tumor alterations may lead to tumor-specific immunity and multiple immune biomarkers are likely candidates for prediction of response to immune-checkpoint therapies.

Interestingly, when focusing on clusters of mutations that predict patient outcome there is growing evidence that immune gene expression is an attractive candidate [76]. Studies in colorectal cancer have shown that there are many common germline mutations among tumors, but neoantigen mutations are distinct between patients [98]. However, when comparing highly mutated tumors to less mutated tumors it was revealed that tumors with more mutations had a histological immune signature consisting of depleted immunosuppressive cells and upregulated immune inhibitory molecules. Inversely, less mutated tumors had amplified immunosuppressive cells, downregulation of HLA molecules, and reduced expression of immune inhibitory molecules. Additionally, the adaptive immune response is highly accurate at predicting patient outcome [76]. This is particularly true for genomic alterations in chemokines and cytokines related to T cell trafficking and homeostasis. The adaptive immune response is shaped by CD8+ T cells, CD4+ T cells, B cells, and follicular helper T cells (Tfh) that help organize lymphoid structures. IL-21 and IL-15 are part of the gamma-chain cytokine family and are crucial for the survival and proliferation of Tfh, CTL, and memory T cells. Consequently, both IL-21 and IL-15 are being used in clinical trials as immunotherapy for cancer.

Identification of the viral, bacterial, or other immunogens that drive the proliferation of these cells could be used to enhance an existing immune response or initiate one that is lacking or absent. It is tempting to speculate that the effect of Coley’s toxin was based not so much on its effect on the innate immune system but rather that it generated adaptive immunity with the ability to cross react and recognize tumors as a result of such mutations to generate tumor regression.

## Peripheral biomarkers prior to treatment

To define the nature of the tumor microenvironment prior to treatment, and its prognostic or predictive value, it is critical to obtain tissues from surgery or from biopsies with sufficient material for immune biomarker analyses. However, this can often be challenging, due to either accessibility, limited size of tumors, and time required for organizing and analyzing tumor tissue collection potentially resulting in significant delays in treatment [99]. Consequently, analysis of readily accessible samples such as peripheral blood is essential for the development of clinically useful biomarkers.

To date, no validated FDA-approved circulatory immunological biomarker exists for patients with cancer, despite technical advances in genomic, proteomic, and metabolomics. Still, biomarkers from peripheral blood would be ideal to provide clinical guidance and to incorporate into routine clinical practice due to accessibility. A number of strategies and techniques have been used to explore the applicability of circulating biomarkers, including the functional status of tumor specific T cells, CD8+ T cell differentiation and sensitivity to apoptosis, levels of circulating immunological mediators, miRNA, and tumor-derived exosomes [100–106]. We explore below strategies in development for immune-related baseline biomarkers of immunotherapy.

### High-dimensional blood profiling of immune cells-can this be a window into the tissue microenvironment?

The impact of immunotherapeutic agents on peripheral blood markers has been documented. For example, during the clinical development of the antibody targeting CTLA-4, ipilimumab, it was quickly identified that CTLA-4 blockade resulted in upregulation of HLA-DR and ICOS by T cells in both the TME and the blood [107–109]. These findings, however, have been primarily shown to be pharmacodynamic markers rather than clinically useful predictive biomarkers for therapeutic decision-making. It is therefore critical to sample the periphery in a high-dimensional manner to look for immune subsets that may be associated with immune fitness at baseline, or to find representative immune actors from the tumor milieu in circulation, for their pro- or anti-tumor activity.

To assess potential pre-existing blood-based cellular biomarkers, fluorescence flow cytometry has become the immunologist’s tool of choice for the analysis of immune cell populations. The technology has become increasingly democratized by the availability of cytometers at reasonable cost. In addition, the wide array of antibodies specific for cell-surface proteins, MHC/peptide multimers and intracellular phosphoproteins and cytokines allows for multiparameter analysis of rare immune cell subsets. While detection of eight markers in a sample is well established for flow cytometry, overlap of the emission spectra of fluorescent antibody labels can present challenges for the analysis of some combinations. The recent development of mass cytometry or cytometry by time-of-flight (CyTOF) for multiparameter single cell analysis, which uses heavy metal ions as antibody labels, overcomes the many limitations of fluorescence-based flow cytometry. CyTOF has very little overlap between channels and no background, allowing for as many as 40 labels per sample. Consequently, CyTOF is being employed to analyze the profile and function of immune populations in a comprehensive manner [110–114].

Efforts are underway to find measurements in blood that may correlate, or at least approximate findings from the tumor tissue site. Clinical examples of correlations between circulating blood and tumor MDSC levels at baseline have been described in several tumor types [115]. Though typically not sufficient to predict outcome alone, peripheral immune markers may be of use in the context of specific immunotherapies, including vaccines. Recently, the concept of “peripheral immunoscore” has been proposed as a predictive baseline biomarker in two different cohorts receiving cancer vaccines [116].

Though rare, tumor neoantigen-specific T cell clonotypes have been identified in the circulation of cancer patients [86, 117, 118]. Moreover, with the growth of adoptive immunotherapy trials, both chimeric antigen receptor and T cell receptor (TCR) transduced T cells that traffic to the tumor and then recirculate are available for analysis. Therefore, tumor-reactive lymphocytes in the circulation can be sampled and interrogated through multiparameter immunophenotypic analysis as a step toward biomarker development. The use of flow cytometry in adoptive cell transfer studies has identified biomarkers associated with persistence, the establishment of antitumor memory, and improved clinical outcomes [119–121]. Recently published observations also confirm that PD-1 expression by peripheral lymphocytes correlates with tumor burden, and the impact of in vivo PD-1 engagement can be measured on circulating T cells and serve as a biomarker for response to immunotherapy [122, 123].

### Immunoprofiling of antigen-stimulated blood, supernatant multiplex analysis and complements in tissue biopsies

Several studies are pointing toward a correlation with peripheral immunological parameters indicative of improved activation or restoration of local tumor immune functions [57, 58, 124, 125]. It would be expected that predictive circulating biomarkers will include markers of increased Type 1 immunity and cytotoxic cell activity, akin to the correlates of improved survival at the tumor site identified in the immunocontexture of cancer [1, 9]. These may include cytokines (including IFNγ, IL-12, IL-2) and chemokines (such as CXCR3 and CCR5 ligands) associated with tumor trafficking, promoting the IFNγ pathway, and stimulating cytotoxic functions [17]. On the other side of the equation, disruption of immunosuppressive pathways characteristic of the TME, such as indoleamine 2,3-dioxygenase (IDO), induction of MDSC, and immunoregulatory pathways may also be relevant.

Multiplex analysis of immunological mediators in blood allows for the rapid quantification of a large range of circulating analytes in small volumes of samples. This approach provides an important source of data to identify predictive biomarkers in cancer patients and direct therapeutic options. Unfortunately, despite extensive use in clinical cancer research over the last decades, no validated results for either diagnosis or prognosis have been obtained. Recently, the NIH/NIAID and the Cancer Immunotherapy Consortium of the Cancer Research Institute have developed a collaboration to monitor proficiency testing in 25 selected sites worldwide and identify variables with the aim to reach standardization of this platform [126]. In the meantime, additional approaches are now being considered, including whole blood collection and stimulation platforms, which may provide further insight and aid in the identification of relevant biomarkers.

A recently developed approach to measure cytokine production from small amounts of blood may provide additional information by capturing peripheral immune cell activity ex vivo. Measures of inflammatory proteomic signatures induced by a variety of immunological stimulants aimed at different cell subsets may yield novel biomarkers with functional relevance. An example of implementation of such analyses in the clinical context is the TruCulture® system, to assess immune cell activity. This syringe-based device is designed to allow for the sterile collection of whole blood and a variety of immunological stimulants aimed at different immune cell subsets [127]. Supernatants, thereby enriched for secreted immunological mediators are quickly obtained with limited manipulation and can be analyzed by multiplex platforms, either using electro-chemiluminescent based immunoassays or bead-based immunoassay technology, readily available to most laboratories. In healthy volunteers, this technique has been used to successfully quantify production of immunological mediators and was shown to differentiate specific proteomic profiles for each of the stimulants used [128, 129] as well as distinguish immune responses to determined treatments [130, 131]. Thus far, data are limited when it comes to patients, but the technique has revealed a distinctive pro-inflammatory signature characterized by altered endothelial cell function and inflammation in schizophrenia [132] and therefore may also be able to provide important clues in circulating immunological parameters in cancer patients.

### T cell receptor diversity in anti-tumor response

T lymphocytes are key players in the anti-tumor response induced by immunotherapies, and defining their repertoire at baseline is a useful tool to determine general immunocompetence and to quantify pre-existing tumor-specific clones. The characterization of T cells has long since focused on protein expression and functional tests. TCR diversity allows for the recognition of a variety of epitopes through TCR-MHC interaction and is associated with the effective control of viral infections, other pathogens [133–135], and tumor cells. TCR diversity is generated by a complex mechanism mainly based on genetic recombination of the DNA resulting in a tremendous range of antigenic specificities. Historically the analysis of TCR diversity has been set aside due to its complexity and to the lack of relevant technologies to accurately measure it. These past few years have seen a growth of interest for the TCR diversity analysis as technology gained precision and robustness. Following the approval of immunotherapies such as anti-CTLA-4 (ipilimumab) and anti-PD-1 (nivolumab and pembrolizumab) in various tumor types, the need for a better understanding of the patient immune system has become even more critical. Accumulating data on TCR diversity has been generated, highlighting its key role in response (clinical benefit and toxicity) to these immunotherapies.

TCR diversity has been estimated from 10^8^ to up to 10^15^, which illustrates how challenging the assessment of such a parameter can be. TCR diversity can be evaluated by NGS, multi-N-plex qPCR, spectratyping and immune phenotyping, each technology having its own depth of analysis and robustness. The NGS approach provides the CDR3 sequence of the TCR, from gDNA or RNA samples [136]. The multi-N-plex qPCR provides an exhaustive semi quantitative assessment of combinatorial diversity (i.e., all V-J rearrangements). It uses genomic DNA and a highly robust and reproducible PCR process, which make it appropriate for routine clinical evaluation of TCR diversity. Spectratyping was the first molecular technology and is based on RNA PCR amplification of V-C combinatorial diversity allowing the distinction of 10 to 13 CDR3 peaks per V gene. This technique is mainly used for basic research. Other method can assess TCR diversity at the protein level, with antibodies, but they have lower resolution, as are limited to the V genes.

Several studies relate the impact of immunotherapies on TCR diversity in peripheral blood. Indeed, it has been shown that CTLA-4 blockade with tremelimumab diversifies the peripheral T cell pool, underscoring the pharmacodynamic effect of this class of immune-modulating antibodies [137]. Cha et al. have demonstrated that CTLA-4 blockade induces T cell repertoire evolution and diversification. Moreover, improved clinical outcomes were shown to be associated with less clonotype loss, consistent with the maintenance of high-frequency TCR clonotypes during treatment [124]. Postow et al. have shown that baseline TCR diversity in the peripheral blood was associated with clinical outcomes [138]. Few results are available regarding the impact of anti-PD-1 on the diversity of TCR. A small study by Robert et al. comparing repertoire evolution under anti-CTLA-4 and anti-PD-1 treatment (9 anti-PD-1 patients; 21 anti-CTLA-4 patients; 4 controls) shows that anti-PD-1 does not diversify the immune repertoire whereas anti-CTLA-4 does [139].

### Prognostic/predictive value of serological markers and B cells in cancer

B cells are commonly found at the tumor site of various malignancies, often organized in germinal centers, resulting in the presence of plasma cells as well. Although their function is still largely unknown, they suggest an ongoing immune reaction at the tissue site. In parallel, circulating autoantibodies with specificity to tumor-derived antigens can often be detected in cancer patients and help identify immunogenic targets in cancer [32]. In general, whether tumor antigen-specific antibodies are by-products of aberrant/ectopic expression or whether they may have a functional role, such as helping cross-present tumor-derived antigens to facilitate T cell recognition [140], there is evidence that detection of IgG specific to tumor associated targets like cancer-testis antigens may act as a surrogate for the presence of T cells [141]. Paradoxically, most of the tumor antigens targeted by known autoantibodies are intracellular, making it more challenging, though not impossible [142], that they would convey a direct antitumor role. In the context of checkpoint blockade, NY-ESO-1-specific autoantibodies have been found to be associated with greater clinical benefit in advanced melanoma patients treated with ipilimumab [143]. This raises the intriguing hypothesis that tumor-specific antibodies may serve as an indicator of the presence of tumor-specific T cells in the tumor microenvironment, where patients with pre-existing capacity to react to tumors may be favorably predisposed to immunomodulatory treatment.

In support of this hypothesis, the presence of highly organized B cell clusters forming germinal centers at the tumor site, with areas including plasma cells surrounded by T cells, and forming tertiary lymphoid structures (TLS) [144], was shown to be highly predictive of outcome in diverse solid tumors, such as NSCLC [145] and melanoma [56]. In particular, the density of B cells as well as mature dendritic cells (DC) correlated with that of TLS in NSCLC, and together were the best predictors of progression-free survival (PFS) and overall survival (OS) in patients following surgical resection [146]. What is the significance of these ectopic lymph node-like structures? Their close proximity to the tumor tissue suggests an active role in local immunogenicity, and there is now evidence that infiltrating B cells as well as T cells have tumor specificity. Following in vitro expansion and differentiation into plasma cells, B cells isolated from NSCLC tumors produce measurable IgG and IgA antibody titers against known tumor associated antigens such as NY-ESO-1, TP53, or XAGE-1 [145]. This further supports the idea that B cells specific for tumor antigens contribute to immune mechanisms present at baseline and represent potential targets for immunotherapeutic intervention.

Whether these immune responses develop first in classical secondary lymphoid organs to eventually reassemble near the antigen source, or originate locally to eventually become systemically detected is yet to be determined. Mice devoid of lymph nodes can still mount an immune response thanks to ad hoc TLS structures in tissues, which suggests the potential for direct priming locally [147]. Linking the presence of these local antibody responses to systemic humoral immunity will also be key in establishing serology as a prognostic or predictive marker of disease outcome.

### Circulating MDSC and suppressive cells

Besides adaptive immune responses (T and B cells), suppressive immune subsets have been proposed as key factors explaining why cancer progresses despite baseline immunity, and why they may be the best targets for immunomodulation. Increased frequency of MDSC in the blood of cancer patients may be an indication of tumor progression, commonly dependent on the tumor stage, at least for some solid and hematologic malignancies. As a consequence of the impact of therapy on tumor mass, MDSC levels can also decrease after treatment and inversely correlate with response to chemotherapy or surgery [37, 148]. However, some data indicate that the frequency of circulating MDSC can be associated with patient prognosis independently of tumor burden [149, 150]. Interestingly, an algorithm for prediction of therapeutic responses to immune checkpoint inhibitors based on MDSC blood frequency was developed and is being tested in clinical trials [150].

Characterization of MDSC is commonly performed by flow cytometry. Different subsets of human MDSC have been described using a combination of myeloid markers, and define three main categories of MDSC. Immature MDSC are positive for the common myeloid marker CD33, but lack the expression of HLA-DR as well as lineage-specific markers of differentiated leukocytes (lin^−^ cocktail usually contains antibodies specific to CD3, CD14, CD16, CD19, CD20, and CD56). More differentiated MDSC are divided into subsets including polymorphonuclear (PMN)-MDSC (CD11b^+^/CD14^−^/CD15^+^/HLA-DR^−^) and monocytic-MDSC (CD11b^+^/CD14^+^/IL4Rα^+^/CD15^−^/HLA-DR^−^) [37, 148]. In most studies a single defined MDSC subset is analyzed, which is a major limitation considering the lack of univocal data about phenotypes and the heterogeneity of human tumors. Unfortunately, only a limited number of studies provide experimental evidence that the analyzed myeloid cells indeed exert an immunosuppressive activity on activated T cells, the main characteristic of MDSC [151]. The first comprehensive immune monitoring of human MDSC employed a nine-color analysis of six subsets of myeloid cells in a randomized, phase II clinical trial in renal cancer patients vaccinated with a multi-epitope mixture of shared cancer antigens [152]. In this study, five out of the six MDSC subsets were significantly expanded in the blood of the 68 monitored renal cancer patients compared to healthy donors. Moreover, the level of two of the MDSC subsets, prior to vaccination, significantly negatively correlated with overall patient survival [152].

A few studies have provided the initial indication that levels of MDSC inversely correlate with OS in metastatic melanoma patients treated with ipilimumab [150, 153] and that a decrease in MDSC after neoadjuvant ipilimumab treatment of patients with melanoma correlates with improved PFS [154]. In view of the immune-mediated mechanisms of action by ipilimumab, lower levels of suppressive cells could represent not only an estimator of the clinical benefit but also a pharmacodynamic biomarker, reflecting the shift from immune escape to immune response. To date, however, there is no evidence to indicate whether ipilimumab targets MDSC directly or, conversely, whether the lower MDSC levels observed following ipilimumab treatment are an indirect outcome of tumor shrinkage in response to immune-mediated rejection. In the future, it will be important to evaluate MDSC as potential biomarkers in patients treated with other immune checkpoint inhibitors (i.e., anti-PD-1/PD-L1) or agonistic antibodies (i.e., anti-CD40).

Given the discrepancies in the field, a proficiency panel for human MDSC was established under the umbrella of the Association for Cancer Immunotherapy immunomonitoring group. In this panel, ten different subsets of MDSC were evaluated simultaneously by 23 experienced laboratories in Europe and the USA, representing the largest MDSC analysis undertaken so far. Analysis of the first phase from this panel is now available, demonstrating variability in MDSC determination, and calling for the harmonization of this field [155]. Moreover, the panel has made recommendations to standardize handling of samples, as subsets such as PMN-MDSC are particularly susceptible to damage from freeze-thawing protocols.

## Predictive biomarkers for adjuvant therapy

Immunotherapies that have shown evidence of antitumor effects in the setting of advanced inoperable disease are now moving to the adjuvant setting, i.e., administered in patients with earlier stages following surgical treatment to reduce the risk of relapse and/or mortality. While we focused so far on baseline biomarkers prior to therapy, surgical tumor resection may be seen as a “reset” of the baseline that warrants the exploration of immune-based biomarkers in patients without evidence of tumor but likely to recur. The prognostic assessment of risk for relapse/mortality is therefore central to the pursuit of adjuvant postoperative therapy, since the candidate for adjuvant therapy must have an elevated risk of recurrence. This relapse risk arises from micrometastatic (clinically undetectable) disease, beyond the scope of the locoregional surgical (or other, e.g., radiotherapy) treatment. The fundamental tenet of adjuvant therapy is thus, that treatment in the adjuvant setting has a therapeutic benefit that exceeds the benefit of later treatment at recurrence, with metastatic disease that is potentially inoperable. Toward this end, biomarkers have been evaluated to ’ refine our assessment of relapse risk and mortality risk and (2) predict the likelihood of benefit (or conversely, toxicity) from therapy.

Melanoma was the first solid tumor for which immunotherapy was successfully pursued, beginning with recombinant IFNα-2a and IFNα-2b in the early 1980s. More than 22 phase III studies have now been completed that show a consistent reduction in relapse risk and improvement in OS with IFNα-2a/b in individual trials, as well as several meta-analyses [156–159]. Unfortunately, only a small subset of these postoperative trials were accompanied by corollary studies designed to identify the mechanism of action for this, and more recently examined candidate adjuvant therapy agents. Broadly useful prognostic and predictive biomarkers were not identified in the gamut of the postoperative phase III trials reported to date. Studies of the peripheral blood obtained at multiple time points during one of the largest of these US Intergroup trials E1694 [160] showed that baseline blood pro-inflammatory cytokine and chemokine levels determined by bead immunoassay correlated with relapse-free survival among patients receiving IFNα-2b, but not an inactive (GM2 vaccine) control. Evaluation of the phenotype of blood lymphocytes has not yielded consistent or useful data, and prospective clinical/serological studies have shown a correlation of the development of autoimmunity with therapeutic benefit in terms of both relapse-free and OS, in the He13A/98 Hellenic Oncology Group trial [161]. The first promising biomarker of antitumor benefit in an adjuvant trial was the clinical and serological evidence of autoimmunity, which correlated with improved PFS and OS (p < 0.01), and was predominantly manifest in autoimmune hyperthyroidism or hypothyroidism [161]. Retrospective serological studies that differed in omitting the clinical assessment of autoimmunity have shown conflicting results [162]. However, the development of clinical and/or serological manifestations of autoimmunity during therapy is a biomarker that cannot be utilized to select patients pre-treatment. Baseline pre-treatment studies of S100 protein levels in the blood (>0.15ug/L) have demonstrated modest prognostic utility, but have had limited application due to variable availability, and the marginal added value [163]. The E4697 Intergroup phase III trial of GM-CSF (Sargramostim, Sanofi) has shown no significant benefit of adjuvant therapy with GM-CSF, alone or combined with a triple lineage antigen peptide vaccine for patients with resectable stage III/IV disease; it also showed no prognostic or predictive utility for the assessment of immunological response to the peptide vaccine by ELISpot [164]. The EORTC 18071 phase III adjuvant trial of high-dose ipilimumab for stage III resected melanoma (10 mg/kg given for 3 years) reported improvement in relapse-free and OS, without corollary immunological or other assessments reported to date [165].

The exploration of the multitude of new immuno-oncology agent combinations for adjuvant therapy of melanoma and other solid tumors demands more efficient approaches than were previously required when therapeutic options were limited. The embarrassment of riches in the advent of >10 new agents for treatment of metastatic melanoma poses issues for the development of combined modality adjuvant therapy that high-throughput bioinformatics, multiplex IHC, and NGS are uniquely able to address.

## Host related biomarkers

After focusing on peripheral and tissue biomarkers, it is important to consider host-related factors that could have a role in general immunocompetence and immunotherapy outcomes, not unlike what is observed for tumor susceptibility in mice of different strains.

### Single nucleotide polymorphisms

SNP represent normal variations in single nucleotides throughout the genome. Some SNP (nonsynonymous) will impact the amino acid sequence of an encoded protein and are responsible for the variations observed in protein sequences. SNP have been linked to the development of different diseases, variable response to drugs, differing toxicities induced by drugs, and the ability to respond to infections. There are an estimated ten million SNP in the human genome found in both coding and non-coding regions. The most common method used to analyze SNP is via commercial SNP array platforms. Most platforms can evaluate up to one million SNP per individual with an accuracy of 99%. Linkage disequilibrium, which is the nonrandom combination of SNP in certain chromosomes, allows commercial platforms to detect 80% of common SNP [166].

GWAS are needed to determine the functional significance of SNP. GWAS attempt to find the variations that are of importance by identifying those that are statistically more prevalent in individuals with one condition compared to individuals without that condition. A challenge in GWAS studies is the large numbers of cases and controls needed for statistical power to obtain extremely low *p*-values. The ability to identify hundreds of thousands of variants causes a multiple testing burden resulting in a high false positive association rate. To have some confidence in the association of a SNP and a particular disease or condition, the *p*-value threshold for significance must be very stringent, i.e., 10^−6^ or lower.

There is significant evidence that SNP play a major role in modulating both levels of immunity and the immune response to different stimuli. Studies have been performed evaluating the phenotype of multiple immune cell subsets and analyzing their variability across a population in association with detected genomic variants [167]. Investigators identified several provocative correlations. A SNP in ENTPD1 which encodes CD39 accounted for 61% of the phenotypic variation in the levels of CD39+ CD4+ Treg. A variant identified near *IL2RA*, a gene encoding the transmembrane portion of CD25, was associated with differing levels of T cells highly expressing CD25. Similarly, a variant near the genes for *CD8A* and *CD8B* was associated with diversity in the levels of T cells expressing CD8. More recent studies have suggested that SNP are critical in CD4+ T cell development and activation especially for Treg and Th17 cells [168]. These data underscore the role of SNP in governing the level and activation state of immune cells.

Genetic variants have been extensively studied as a cause for the diversity seen in the ability to generate an immune response after vaccination or even the level of immunity achieved after vaccination. Two frequent examples are variations in MHC genes as well as genes encoding cytokines or associated with cytokine secretion. Investigators have demonstrated that SNP occurring within MHC class I and II genes were correlated with response to childhood vaccinations [169]. Specific SNP in MHC genes were not only associated with serum levels of immunoglobulins and isotypes but also with the variations observed in vaccine-specific antibody responses generated with immunization. A recent meta-analysis evaluated 13 GWAS including over 11,000 individuals who were immunized with common vaccines. Seven SNP in HLA genes were included in the analysis and significant associations were found for SNP that were linked with significant decreases in antibody responses (DRB1*07, DQA1*02:01, DQB1*02:01, and DQB1*03:03) and SNP that were associated with a significant increase in antibody response with vaccination (DRB1*13 and DRB1*13:01). Studies of measles and rubella vaccines suggest that SNP linked with secreted IL-6 and IFNγ may dictate variations in the levels of the vaccinated immune response observed between individuals [170, 171]. The studies described above demonstrate just a few examples of immune associated SNP, although many more have been identified. For example, responses to vaccines or monoclonal antibody therapy have been related to SNP in Fc receptor genes or genes associated with innate immune cells [172, 173]. Additionally, specific SNP in toll-like receptor genes have been associated with disease [174].

Most immuno-oncology trials have not focused on an evaluation of SNP as a cause of clinical response diversity, lack of response, or variations in immunity. There are sufficient data in the literature to begin to validate the most well studied immune related SNP as a cause of response diversity.

## Conclusions

Tumor cells do not grow and survive in isolation but rather interact with intratumoral immune cells. Consequently, this immune interaction with the underlying tumor immunome and the TME determines tumor survival [76, 98]. The recent success of immunotherapies targeting the immune checkpoint molecules, CTLA-4, PD-1, and PD-L1 for the treatment of cancer has emphasized the essential role of the immune system for eradicating tumors. While these immunotherapies have had stunning results, the percentage of patients with clinical benefit is limited and the reason behind this is not well understood. The ability to predict whether a patient will respond or become resistant to immunotherapy is essential to finding a cure for cancer.

Pairing clinical response data with an interrogation of the TME and circulating immune indicators that can serve as a window into the TME will be critically important to identify relevant biomarkers.

One of the key factors that may contribute to a better understanding of the impact of immunotherapies on the patient adaptive immune system appears to be TCR diversity. Additional clinical assessment and validation (both retrospectively and prospectively) are ongoing to confirm the relevance of TCR diversity (in blood or at tumor site), alone or in combination with other immune parameters, to predict response to cancer immunotherapy. Diligent sample (blood/PBMC/tumor/lymph node) and data (including clinical response according to immune related Response Criteria) [175] collection in ongoing and future cancer immunotherapy clinical trials will be critical to achieve this goal. Alternatively, measurement of tumor-infiltrating B cell responses may present some advantages as the mark of “local immunocompetence” because measurement of antibodies can be performed in high-throughput with greater ease compared to T cell specificity assays. There is a clear need in the future to use multiplex IHC to characterize the TME beyond just T cells to include B cells and markers for TLS as well. While TCR sequencing has led to useful information about clonality and diversity of the repertoire, it would also be of interest to quantify the changes in B cell repertoire at the tumor site in light of their presence and prognostic role in tissues. Eventually, a need to develop predictive methods to link B cell receptor sequences with specificity to antigens would provide the greatest leap forward.

A powerful approach to integrate the value seen in studies of both T and B cells in the setting of malignant solid tumors is gene-based immune diagnostics. Perhaps the greatest challenge facing the development of gene-based immune diagnostics is the lack of data comparing the prognostic and predictive qualities of immune genes and gene signatures to that of gold standard IHC-based methods for quantifying immune cell abundance and functional orientation. From a logical perspective, the greatest potential for immune gene signatures may be found in the prediction of responsiveness to current and emerging immune therapies. In this context, patient cohorts randomized to treatment with sufficiently large n and longitudinal endpoints encompassing both tumor response and patient survival will be essential for comprehensive assessment of clinical utility. Furthermore, to enable rigorous comparisons, standardized protocols for the histopathological assessment of TIL and effector cell populations will need to be developed and uniformly implemented, as discussed [49, 176, 177]. Finally, emerging evidence from breast cancer studies indicating that immune gene classifiers of outcome exhibit significant associations in some cancer subtypes, but not others, suggest that heterogeneity related to tumor immunogenicity, mechanisms of immune tolerance, or other factors that influence immune function may need to be accounted for to determine the applicability of immune diagnostics for individual patients.

Toward the goal of defining the role of local innate immune cells in the TME, an international proficiency panel for human MDSC has made strides toward assay harmonization in order to address discrepancies in the field. This panel also agreed upon recommendations for standardization of sample handling. However, MDSC characterization as biomarkers might benefit further from a number of additional analyses. Molecular markers associated with effector inhibitory mechanisms (*ARG1, NOS2, IDO1, IDO2, NOX2, PD-L1, PD-L2, IL-10*) could, at least in theory, avoid the cumbersome and difficult to standardize functional studies. In addition, comparison between circulating and tumor-associated myeloid cells in each single patient, both before and after immunotherapy, might help to address the issue of the cross-talk between local and distant tumor-conditioned environments and rank the usefulness of the relative biomarkers.

The prediction of therapeutic benefit from immunotherapies presumes knowledge of the mechanism of action that has often not been available. The advent of new technologies has made possible a more comprehensive analysis of the immune system in the TME, which will yield valuable mechanistic data that can be translated into clinically relevant biomarkers. A depth of understanding of the relationship between pre-existing immunity and the TME is now more important than ever, as approvals for new combination and adjuvant therapies add a layer of complexity to this dynamic puzzle.

In conclusion, experts from Working Group 4 of SITC’s Immune Biomarkers Task Force have explored in this manuscript several facets of what contributes to baseline immunity against tumors and that may predict clinical outcome in cancer patients. In contrast to other Task Force initiatives, the recommendations made here are more exploratory, as this is a nascent but rapidly evolving topic. Continuing discoveries in host genetic factors (SNP), tumor alterations in genes and proteins affecting the antigen presentation machinery [178, 179], or the local recruitment of immune actors [180, 181] all contribute to our understanding of how the TME becomes organized and affects peripheral immune detection in the circulation. While immunocompetence is still difficult to define as a biomarker, it is likely that a combination of personalized measurements will be required for an accurate correlative predictive signature in each patient.

## Abbreviations

CTL: Cytotoxic T lymphocyte(s)
CTLA-4: Cytotoxic T lymphocyte-associated protein 4
CyTOF: Cytometry by time-of-flight
DC: Dendritic cell(s)
DFS: Disease-free survival
FDA: Food and Drug Administration
GWAS: Genome-wide association study
IDO: Indoleamine 2,3-dioxygenase
IHC: Immunohistochemistry
IVDMIA: In vitro diagnostic multivariate index assay
MDSC: Myeloid-derived suppressor cell(s)
MHC: Major histocompatibility complex
MSI: Microsatellite instability
NGS: Next-generation sequencing
NK: Natural killer
NSCLC: Non-small cell lung cancer
OS: Overall survival
PD-1: Programmed cell death protein 1
PD-L1: Programmed death ligand 1
PFS: Progression-free survival
PMN: Polymorphonuclear
SITC: Society for Immunotherapy of Cancer
SNP: Single nucleotide polymorphism(s)
TCR: T cell receptor
Tfh: Follicular helper T cell(s)
Th: Helper T cell(s)
TIL: Tumor-infiltrating lymphocyte(s)
TLS: Tertiary lymphoid structure(s)
TME: Tumor microenvironment
Treg: Regulatory T cell(s)
